# PTEN and p53 Combined Gene Therapy Promote Apoptosis and Chemosensitivity to Oxaliplatin in Colorectal Cancer: An *In Vitro* Study

**DOI:** 10.34172/apb.43371

**Published:** 2025-02-09

**Authors:** Narjes Nakhaee, Sirous Zeinali, Mahboubeh Kabiri, Ladan Teimoori-Toolabi

**Affiliations:** ^1^Department of Biotechnology, Faculty of Science, Tehran University, Tehran, Iran.; ^2^Molecular Medicine Department, Pasteur Institute of Iran, Tehran, Iran.

**Keywords:** Gene therapy, Tumor suppressor, PTEN, P53, Colorectal cancer

## Abstract

**Purpose:**

Cancer is a complex condition and gene therapy has evolved as a promising method for cancer treatment. Studies have demonstrated that PTEN and p53 proteins have remarkable antitumor effects but combined up-regulation of both PTEN and p53 genes has not been reported. We thus investigated their therapeutic potential in colorectal cancer (CRC) cells.

**Methods:**

PTEN, p53, and blank vectors were purchased from Addgene, and transfected in SW480 cell line. Cell viability and apoptosis was assayed by MTT and flow cytometric analysis respectively. Real-time PCR assay was applied to assess changes in the expression of genes. To evaluate the effect on drug sensitivity of transfected cells, flow cytometric analysis was conducted.

**Results:**

PTEN are more able to induce apoptosis than p53 in SW480 and PTEN and p53 demonstrated a synergistic anticancer impact. Further tests showed that both genes increased the change in the expression of genes related to cell cycle and apoptotic factors. Co-expression of these genes can also increase the susceptibility of CRC cells to the chemotherapeutic agent oxaliplatin.

**Conclusion:**

According to our findings, cancer gene therapy targeting two tumor suppressors, like PTEN and p53 genes, might be a potent therapeutic approach for treating colorectal and other cancers.

## Introduction

 Colorectal cancer (CRC) is a prevalent malignancy affecting both women and men. It is the second most common cause of cancer related deaths, with over 100 000 new cases and 50 000 deaths reported in the United States in 2021.^1^ Despite advances in surgical techniques, the success rate for CRC treatment remains unsatisfactory, due to the disease’s rapid invasion and earlymetastases.^2^ Systematic treatment is therefore essential for advanced casesthat are ineligible for surgery. However, the frequent use of chemotherapeutic agents often leads to the development of treatment resistance.^3^ Currently, oxaliplatin-based chemotherapy is the primary approach for treating CRC, but patients eventually relapse due to drug resistance, but patients eventually relapse due to drug resistance.^3^ The primary mechanism behind oxaliplatin resistance remains unclear, posing a significant challenge in CRC treatment. Consequently, it is crucial to develop new combinations of traditional anticancer medications and to elucidate the mechanismsunderlying treatment resistance.

 The development of genetic flaws that cause unchecked cellular proliferation underlies cancer. p53, a 53 kDa protein that controls cell cycle progression and fate, is one of the most extensively researched tumor suppressors. Both growth arrest and apoptosis are triggered upon p53 activation, thus, the inactivation of p53 signaling pathways allows damaged cells to proliferate, leading to the development of tumors.^4^ More than 50% of human cancer have a mutant p53 gene.^5^ Delivery of the wild-type p53 (wt-p53) gene to a cancer cell can increase the levels of wt-p53 protein and trigger apoptosis or growth arrest, leading to the inhibition of tumor growth.^6^

 Another well-studied gene in cancer is phosphatase and tensin homolog (PTEN) a tumor-suppressor gene located on the long arm of chromosome 10. PTEN undergoes frequent deletion and somatic mutation in endometrial cancer, glioblastoma, prostate cancer, and small-cell lung cancer.^7-10^ The development of CRC is closely associated with PTEN status. Severe PTEN deficiency is linked to resistance to treatment, particularly to targeted therapies involving the receptor tyrosine kinases (RTKs) pathway like trastuzumab, and advanced tumor stage.^11^ Targeting the PI3K/ AKT/ PTEN/ mTOR pathway increases patient survival by restoring drug sensitivity in cancer stem cells (CSCs). This system is involved in several mechanism contributing to chemo- and radio-resistance.^12^ Ectopic expression of PTEN causes cell death and growth inhibition in nonsmall cell lung,^13^ colorectal,^14^ and thyroid cancer cells^15^ with wt-PTEN.

 PTEN and p53 tumor suppressors are among the most commonly mutated or inactivated genes in human cancers. Although PTEN and p53 have different functions, it has been suggested that they work in tandem because PTEN regulates p53 stability and p53 is believed to enhance PTEN transcription.^16^ A cross-talk between p53 and PTEN occurs at the transcriptional as well as protein levels, and it is frequently association with mutually antagonist pathways, which often include MDM2.^16^ By suppressing MDM2 transcription and decreasing its binding activity to p53, PTEN increases both the level and activity of p53.^17^ Additionally, PTEN collaborates with the tumor suppressor p53 during oxidative stress, leading to cell cycle arrest.^18^

 The effects of p53 and PTEN have been studied in various cancers separately, but their synergistic effect in suppressing tumors remains unknown. We assessed whether combining p53 and PTEN gene therapy could produce added antitumor effects in human colorectal cell line (SW480), and also examined its impact on oxaliplatin resistance.

## Materials and Methods

###  Cell culture 

 The SW480 cell line was obtained from the National Cell Bank of Iran (NCBI, Pasteur Institute of Iran, Iran). The cells were cultured in high-glucose DMEM/F-12 medium supplemented with 1% penicillin/streptomycin and 10% fetal bovine serum (FBS) (all sourced from Thermo Fisher Scientific Inc., USA). The cultures were maintained at 37°C in a humidified incubator with 5% CO2, and the flask media were refreshed every 2–3 days.

###  Transient transfection

 PTEN, p53, and blank vectors were obtained from Addgene (USA) (pCMV-Neo-Bam p53 wt, Plasmid #16434; pCMV Flag WT-PTEN, Plasmid #22231; pCMV-Neo-Bam, Plasmid #16440). PTEN was subcloned into the pcDNA3.1 vector containing a hygromycin resistance gene.

 For transfection, SW480 cells were seeded in 6-well plates, and transfection was performed once the cell confluency reached 80%. The calcium phosphate method was used for transfection. Two to three hours prior to transfection, the culture medium was replaced with fresh medium. A total of 3–5 **μg** of each vector was mixed with 16 **μL** of 2M CaCl₂ buffer and diluted to a final volume of 125 **μL**. This solution was thoroughly mixed and combined with 125 **μL** of HBS2X solution, then vortexed. The resulting mixture was allowed to sit at room temperature for 20 minutes before being added dropwise onto the cell plates. The cells were incubated overnight at 37°C in a CO₂ incubator.

 After incubation, the medium was replaced with fresh medium containing 10% FBS. To select for successfully transfected cells, the medium was replaced 24 hours later with selective media containing 100 **μg**/mL hygromycin for PTEN-transfected cells or 50 **μg**/mL neomycin for p53-transfected cells. For cells co-transfected with PTEN and p53, the medium contained both hygromycin (100 **μg**/mL) and neomycin (50 **μg**/mL) (Sigma, USA). Following another 24-hour incubation, the medium was refreshed and cells were prepared for further analyses.

###  Cell viability assay by MTT 

 SW480 cell lines in the logarithmic growth phase were cultured in serum-free DMEM medium. Approximately 1 × 10⁴ SW480 cells were seeded into each well of a 96-well plate. The cells were divided into four experimental groups: blank (control), PTEN, p53, and PTEN + p53. These groups were transfected with blank vectors, PTEN plasmids, p53 plasmids, and co-transfected with PTEN and p53 plasmids, respectively.

 After 48 hours of incubation with fresh medium, 20 μL of MTT solution was added to each well. Following a 4-hour incubation, the culture medium was carefully discarded, and 150 μL of DMSO was added to each well. The plates were gently agitated at low speed for 10 minutes to ensure complete dissolution of the resulting violet formazan crystals.

 The optical density (OD) was measured using an ELISA reader at a wavelength of 490 nm. Untreated cells served as the reference group, and the OD values of treated cells were normalized against those of the reference group to assess relative cell viability.

###  Cell apoptosis assay by flow cytometry

 Flow cytometric (FCM) analysis was performed to assess apoptosis in the treated SW480 cells. Cells were transfected with PTEN, p53, PTEN + p53, or a blank plasmid as the control group. Following transfection, cells were stained with Annexin V-FITC and propidium iodide (PI) to evaluate apoptosis and necrosis according to the manufacturer’s protocol.

 Briefly, SW480 cells were incubated for 48 hours post-transfection before being harvested. The harvested cells were washed twice with cold PBS and resuspended in PBS at a concentration of 1 × 10⁶ cells per 100 μL. To this suspension, 1 μL each of PI and Annexin V-FITC was added. The cells were incubated for 15 minutes at room temperature in the dark to ensure proper staining. Apoptosis and cell cycle status were then analyzed using flow cytometry.

 Cells stained with only Annexin V-FITC were classified as being in the early apoptosis phase, while cells positive for both Annexin V-FITC and PI were identified as undergoing primary necrosis or in the late apoptosis stage. This methodology provided a comprehensive analysis of the apoptotic and necrotic states of the treated cells.

###  Real-time PCR assay 

 Real-time PCR was employed to quantitatively investigate alterations in gene expression related to the cell cycle and apoptosis. Total RNA was extracted from transfected SW480 cells using RNX-Plus Solution (SinaClon BioScience, Iran), and mRNA was reverse-transcribed into cDNA using a cDNA Synthesis Kit (Yekta Tajhiz Azma, Iran).

 Primer pairs for quantitative real-time PCR were designed using the Primer-BLAST tool (http://www.ncbi.nlm.nih.gov/primer-blast), and additional analysis with oligo design tools (Generunner 6.0) ensured the exclusion of primers with stable secondary structures. The primers were synthesized by Metabion (Metabion, Germany). GAPDH (glyceraldehyde-3-phosphate dehydrogenase) was used as the reference gene for normalization. Gene expression analysis included GAPDH, BAX, Bcl-2, Caspase 8, Caspase 9, CDKN2A, p53, and PTEN, using the SYBR Green Master Mix.

 Each reaction was conducted in a 25 μL volume in triplicate, comprising 2 μL of cDNA, 12.5 μL of RealQ Plus 2x Master Mix (Amplicon, UK), and 0.2 μM of both forward and reverse primers. Amplification was carried out on a Rotor-Gene 6000 system (Corbet Life Sciences, Australia) with the following thermal cycling conditions: an initial denaturation step at 95 °C for 15 minutes, followed by 40 cycles of 95 °C for 15 seconds, 61 °C for 30 seconds, and 72 °C for 15 seconds. Results were analyzed using REST 2009 V2.0.13 software.

###  Drug sensitivity

 To evaluate the impact of PTEN and/or p53 on the drug sensitivity of SW480 cells, the cells were transfected as described earlier. After 48 hours of incubation, the transfected cells were treated with oxaliplatin at a final concentration of 120 μg/mL (Sigma-Aldrich, USA) for 24 hours. Following the treatment, apoptosis was assessed using flow cytometric analysis to determine the effects of PTEN and p53 on the chemotherapeutic response.

## Results

###  p53 and PTEN significantly suppress the proliferation of SW480 cells

**Figure 1:**
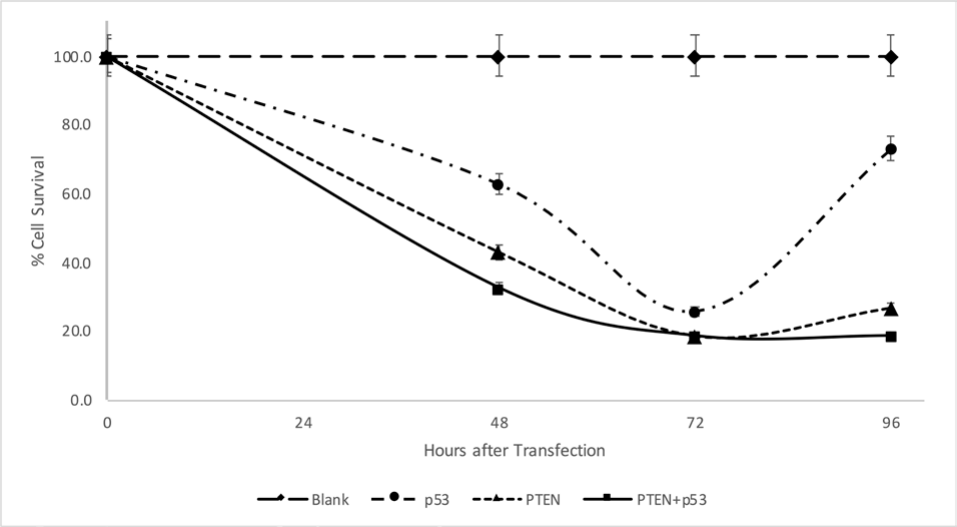
Reduction in cell viability by PTEN and p53 overexpression. MTT assays were performed to evaluate the effect of PTEN and p53 overexpression in SW480 cells 48, 72, and 96 hours after transfection. Experiments were performed in triplicate and error bars depict the standard error of the mean

 To investigate the effect of p53 and PTEN tumor suppressors on CRC *in vitro*, the MTT assay was used to measure the proliferation of SW480 cells. According to Figure 1, compared to control cells that were transfected with a blank plasmid, the viability of the PTEN or p53-transfected cells was significantly reduced. This demonstrates their role as tumor suppressors in SW480 cells by inhibiting the proliferation of tumor cells time-dependently, with a peak suppression occurring 72 hours after transfection (25.6%, 18.5%, and 18% for p53, PTEN, and PTEN + p53, respectively). Co-transfecting p53 and PTEN resulted in a significantly enhanced inhibition of cell proliferation 48 h after transfection compared to overexpressing either p53 (*P* value = 0.009) or PTEN (*P* value = 0.081) alone (62.7%, 42.9%, and 32.9% for p53, PTEN, and PTEN + p53, respectively). This suggests that the co-transfection of p53 and PTEN enhanced the antitumor activity in SW480 cells.

 After 96 hours, an increase in cell survival was observed due to decreased gene expression brought on by plasmid transient transfection (73%, 26.7%, and 33.8% for p53, PTEN, and PTEN + p53, respectively).

###  PTEN dramatically increases the rate of apoptosis compared to p53

 To investigate the effects of PTEN and p53 on inhibiting tumor cell growth, an apoptosis assay was performed on SW480 tumor cells transfected with PTEN, p53, PTEN + p53, and blank plasmids, using Annexin V-PE/7-AAD double staining and flow cytometry. Untransfected cells were included as a control group.

**Figure 2:**
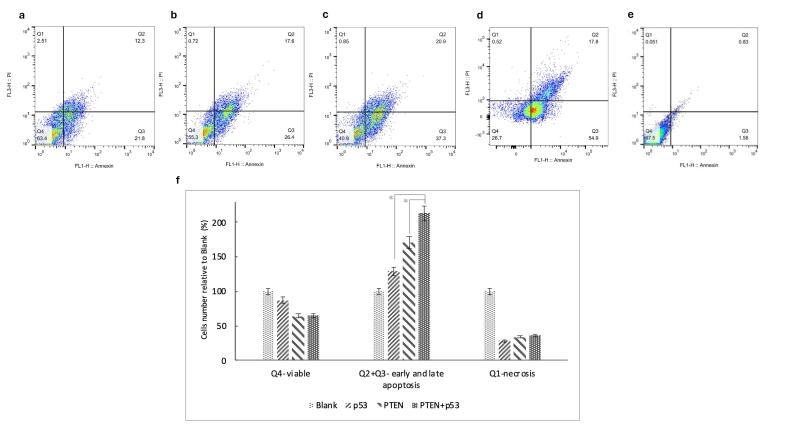
PTEN and p53 promote apoptosis in SW480 cells. Flow cytometric analysis of *in vitro *apoptosis and representative flow cytometric images are shown for cells transfected with blank (a), p53 (b), PTEN (c), co-transfect with PTEN and p53 (d) and unstained controls. The proportion of cells in each quadrant is divided by the corresponding quadrant in the blank group to compare across test groups (f). The asterisks indicate the groups which were significantly different (p < 0.05) from each other. Experiments were performed in triplicate and error bars depict the standard error of the mean

 As shown in Figure 2, compared to control cells transfected with the blank plasmid, transfection with p53, PTEN, or PTEN + p53 resulted in apoptosis rates of 4%, 34%, and 33%, respectively, including both early and late apoptotic cells. Additionally, the necrotic fraction increased significantly in the samples, with necrosis levels 50%, 43%, and 200% higher in cells transfected with p53, PTEN, and PTEN + p53, respectively, compared to normal conditions.

 The findings indicate that apoptosis is significantly induced by PTEN, as observed in both the PTEN (*P* value = 0.02) and PTEN + p53 (*P* value = 0.01) groups. Moreover, co-transfection with PTEN and p53 further increased the overall rate of cell death, demonstrating a 200% increase in cell death relative to control conditions. These results highlight the synergistic role of PTEN and p53 in promoting apoptosis and inhibiting tumor cell survival.

###  PTEN and p53 regulate the expression of cell cycle and apoptosis-related proteins

**Figure 3:**
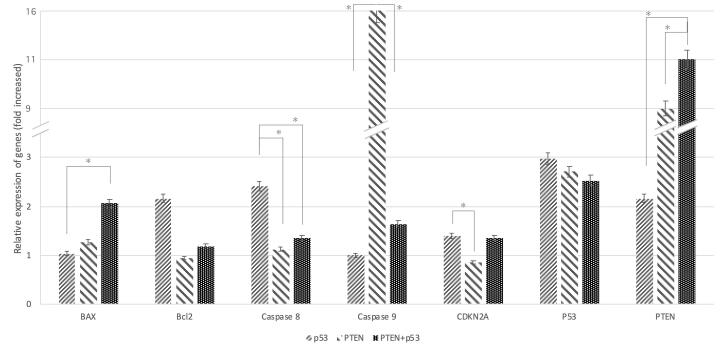
Increased levels of PTEN and p53 trigger the intrinsic and extrinsic apoptotic pathways. Analysis of gene expression associated with apoptosis and normalization against GAPDH expression were conducted. The asterisks indicate the groups which were significantly different (p < 0.05) from each other. Experiments were performed in triplicate and error bars depict the standard error of the mean

 To evaluate the anticancer activity’s mechanism by p53 and PTEN, real-time analysis was used to measure the Transcription level of apoptotic markers and cell cycle-related genes i.e., BAX, Bcl-2, caspase 8, caspase 9, and CDKN2A (Figure 3). The results revealed that transfection with PTEN or PTEN + p53 significantly increased the BAX/Bcl-2 expression ratio (*P* value = 0.02 and 0.03 respectively), indicating a heightened susceptibility to apoptosis compared to p53 transfection alone. Furthermore, p53 transfection led to a marked overexpression of caspase 8, a key mediator of the extrinsic apoptotic pathway, thereby amplifying apoptotic signaling through this route. In contrast, PTEN transfection caused a fivefold overexpression of caspase 9, highlighting its pivotal role in activating the intrinsic apoptotic pathway.

 As expected, co-transfection with PTEN and p53 activated both intrinsic and extrinsic apoptotic pathways, as evidenced by the simultaneous upregulation of caspase 9 and caspase 8. Additionally, p53-transfected cells demonstrated significant overexpression of the CDKN2A gene (*P* value = 0.023), which encodes the p14 and p16 proteins. These proteins are essential for inducing cell cycle arrest, further inhibiting tumor progression. The upregulation of CDKN2A products also amplified apoptosis signals, reinforcing the synergistic effects of PTEN and p53 in regulating cell death and halting cell proliferation.

###  The oxaliplatin resistance of CRC is partly reversible by PTEN and p53

**Figure 4:**
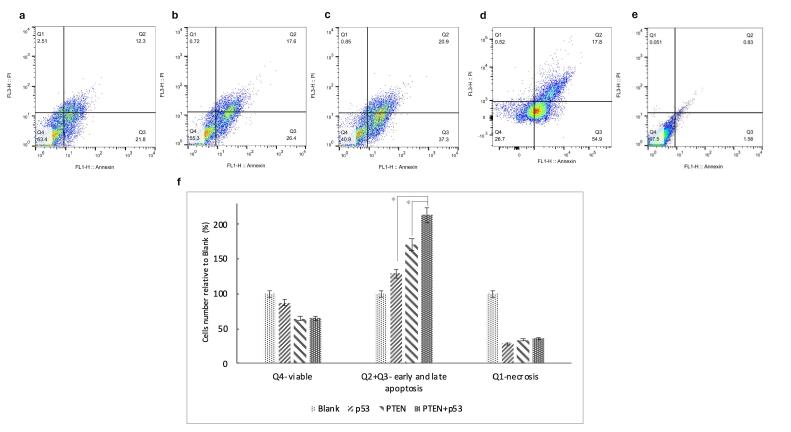
PTEN and p53 enhance sensitivity to oxaliplatin in SW480 cells. Representative flow cytometric images are shown for cells treated with oxaliplatin and transfected with blank (a), p53 (b), PTEN (c), co-transfect with PTEN and p53 (d) and unstained controls(e). The proportion of cells in each quadrant is divided by the corresponding quadrant in the blank group to compare across test groups (f). The asterisks indicate the groups which show a significant difference (p-value < 0.05). Experiments were performed in triplicate and error bars depict the standard error of the mean

 We examined whether co-treatment with PTEN and p53 could influence oxaliplatin resistance in SW480 CRC cells. Flow cytometry assay results demonstrated that transfection with PTEN and/or p53 significantly enhanced the sensitivity of SW480 cells to oxaliplatin treatment (with p-value of 0.012 for PTEN, 0.03 for p53 and 0.014 for PTEN + p53 transfected cells) (Figure 4). Specifically, co-treatment with p53, PTEN, or PTEN + p53 alongside oxaliplatin increased apoptosis by 30%, 70%, and 200%, respectively, compared to cells transfected with the blank plasmid and treated with oxaliplatin. These findings highlight the potential of PTEN and p53 co-expression to overcome chemoresistance and enhance the efficacy of oxaliplatin in CRC therapy.

## Discussion

 Cancer is the focus of over 60% of all active clinical gene therapy trials worldwide, underscoring its critical importance in current biomedical research. Gene therapy has emerged as a promising and innovative strategy for treating a wide range of diseases, particularly cancer, by directly targeting genetic mutations or defective genes that drive tumorigenesis. Among the most effective targets for such therapies are tumor-suppressor genes, which are ideal candidates due to their ability to inhibit tumor growth and induce apoptosis. Studies have demonstrated that targeting ING4,^19,20^ PTEN,^21^ p21,^22^ most prominently, p53 can significantly reduce cancer cell viability. These genes play pivotal roles in promoting tumor cell death, halting proliferation, and limiting cancer progression through regulation of the cell cycle.^23^

 The p53 gene, frequently mutated in cancers, has been a widely studied target in cancer gene therapy since 1994.^24^ Numerous studies have evaluated the effects of p53 gene therapy across various cancer types. Adenovirus-associated p53 gene therapy products, such as Gendicine and Advexin, are currently used in clinical treatments.^25^

 Loss of PTEN activity results in persistent activation of PI3K/AKT signaling, leading to abnormal cell growth, survival, and proliferation.^26^ Research has increasingly focused on the PTEN gene and its downstream signaling pathways, such as PI3K/AKT, to evaluate the effectiveness of gene therapy in treating cancers like prostate cancer,^21^ CRC,^14^ hepatocellular carcinoma^19^ andAcute Lymphoblastic Leukemia.^27^ Mutations in genes like p53 and PTEN have been identified as high-penetrance susceptibility factors and hold significant clinical importance in assessing cancer risk.

 In this study, we investigated the outcome of co-transfecting PTEN and p53 tumor suppressors in SW480 CRC cells in vitro using recombinant CMV-plasmids carrying wild-type PTEN and p53 genes. The combined expression of PTEN and p53 resulted in synergistic apoptosis and growth inhibition in SW480 cells. Similar data have been reported in other studieswhere co-transfection of two tumor suppressor genes led to a notable increase in tumor cell death For example, co-transfection of p53 and ING4 in breast cancer,^20^ PTEN and ING4 in hepatocellular carcinoma^19^ and p53 and p33 in gliomas.^28^ Notably, PTEN induced apoptosis more effectively than p53, as demonstrated by MTT and flow cytometry assays, where PTEN led to 70% higher cell death and 30% more apoptosis compared to p53.

 To understand the synergistic effect of PTEN and p53 co transfection, we must examine how the interact in cell cycle and apoptosis pathway. In normal cells, p53 levels are controlled by MDM2, which itself is regulated by p53 through a transcriptional feedback loop, creating an autoregulatory negative-feedback mechanism.^29^ Under oncogenic stress, the stabilization of p53 is primarily facilitated by the tumor suppressor ARF, which disrupts MDM2-mediated ubiquitination of p53.^30^ Meanwhile, PTEN influences MDM2 at the transcriptional level by regulating its promoter activity and isoform selection. Through these mechanisms, PTEN reduces MDM2 transcription and binding activity, thereby increasing both the levels and activity of p53.^31^

 Another perspective is that PTEN’s ability to dephosphorylate PIP3 to PIP2 inhibits AKT-regulated downstream signaling events, thereby suppressing the PI3K/AKT pathway^32^ which is one of the most frequently activated pathways in human cancers. p53 interacts with this pathway by activating PTEN transcription.^33^ This shows that an increase in both p53 and PTEN protein level in cell can create a positive feedback loop, helping to explain the synergistic effect of co-transfection.

 Real-time PCR analysis revealed the expression of cell cycle and apoptosis-related markers such as BAX, Bcl-2, caspase 8, caspase 9, and CDKN2A. CDKN2A and pro-apoptotic proteins like BAX, which are well-characterized p53 target genes, are key players in apoptosis and cell cycle arrest.^34^ Bcl-2 family is a significant driver of cell fate and a fundamental regulator of apoptosis.^35^

 In our study, caspase 8 and CDKN2A levels were significantly upregulated in p53-treated cells, while PTEN increased caspase 9 expression, highlighting its role in intrinsic apoptosis. Combined PTEN and p53 treatment elevated BAX, caspase 9, and CDKN2A expression, indicating activation of both intrinsic and extrinsic apoptotic pathways. This synergistic effect underscores the enhanced anticancer activity of PTEN and p53 co-expression in CRC cells. Similar studies have been conducted, examining BAX and Bcl-2 expression as well.^19,20^

 Additionally, we observed that PTEN and p53 gene therapy significantly increased the chemosensitivity of SW480 CRC cells, with co-transfection of PTEN + p53 notably enhancing sensitivity to oxaliplatin. Mutations in p53 are linked to diminished responses to various chemotherapeutic agents, including 5-fluorouracil (5-FU), cisplatin, temozolomide, doxorubicin, and gemcitabine, as well as the anti-EGFR monoclonal antibody cetuximab.^36^ PTEN also influences Cyclic AMP-Responsive Element-Binding Protein (CREB) activity, which affects its ability to regulate the transcription of specific genes, thereby modulating sensitivity to chemotherapeutic drugs. Additionally, elevated Bcl-2 transcription has been observed in certain drug-resistant breast cancer cells expressing activated Raf-1.^37^ Activated CREB can regulate Bcl-2 expression, and the interplay between Akt, CREB, and Bcl-2 is implicated in the development of drug resistance.^38^ We discovered that overexpressing PTEN and p53 genes can improve the oxaliplatin sensitivity of SW480 cells and notably, PTEN has more impact than p53 because it directly inhibits the PI3/AKT pathway.

 In conclusion, PTEN and p53 co-expression synergistically induce an enhanced effect on growth suppression, drug sensitivity, and apoptosis stimulation in CRC cells. Cancer gene therapy with a combination of two or more tumor suppressors, like PTEN and p53, can be a promising strategy for CRC treatment.

## Conclusion

 Our overall results strongly suggest that the co-expression of PTEN and p53 tumor suppressor genes exerts a synergistic anticancer effect in colorectal cancer (CRC) cells. PTEN alone induced greater apoptosis than p53, and their combined transfection significantly enhanced apoptotic signaling via both intrinsic and extrinsic pathways. This co-expression upregulated key markers such as BAX, caspase 8, caspase 9, and CDKN2A, contributing to effective cell cycle arrest and programmed cell death. Moreover, co-transfection increased the sensitivity of CRC cells to oxaliplatin, overcoming limitations caused by p53-related chemoresistance. These findings align with previous studies on dual tumor suppressor strategies in various cancers. Based on our results, the combined gene therapy of PTEN and p53 presents a promising approach for enhancing CRC treatment outcomes. However, further in vivo studies are warranted to confirm these findings and assess clinical applicability.

## Competing Interests

 The authors declare that they have no conflict of interest.

## Ethical Approval

 Not applicable.
